# Iodide Analogs of Arsenoplatins—Potential Drug Candidates for Triple Negative Breast Cancers

**DOI:** 10.3390/molecules26175421

**Published:** 2021-09-06

**Authors:** Ðenana Miodragović, Wenan Qiang, Zohra Sattar Waxali, Željko Vitnik, Vesna Vitnik, Yi Yang, Annie Farrell, Matthew Martin, Justin Ren, Thomas V. O’Halloran

**Affiliations:** 1 Department of Chemistry, Northeastern Illinois University, 5500 St. Louis Ave, Chicago, IL 60625, USA; d-miodragovic@neiu.edu (Ð.M.); M-Martin11@neiu.edu (M.M.); 2 Chemistry of Life Processes Institute, Northwestern University, 2170 Campus Drive, Evanston, IL 60208, USA; w-qiang@northwestern.edu (W.Q.); zohrasattar2023@u.northwestern.edu (Z.S.W.); y-yang2@northwestern.edu (Y.Y.); Justinren2022@u.northwestern.edu (J.R.); 3 Division of Reproductive Science in Medicine, Department of Obstetrics and Gynecology, Feinberg School of Medicine, Northwestern University, 303 East Superior Street, Chicago, IL 60611, USA; 4 Institute of Chemistry, Technology and Metallurgy, University of Belgrade, Njegoševa 12, 11000 Belgrade, Serbia; zeljko.vitnik@ihtm.bg.ac.rs (Ž.V.); vesna.vitnik@ihtm.bg.ac.rs (V.V.); 5 Department of Chemistry, University of Illinois at Urbana Champaign, 102 N. Neil St., Champaign, IL 61820, USA; afritz3@illinois.edu; 6 Department of Chemistry and Department of Microbiology & Molecular Genetics, Michigan State University, 567 Wilson Rd., East Lansing, MI 48824, USA

**Keywords:** arsenoplatin, cisplatin, arsenic trioxide, X-ray structure, DFT, antiproliferative activity, triple negative breast cancers

## Abstract

Patients with triple negative breast cancers (TNBCs)—highly aggressive tumors that do not express estrogen, progesterone, and human epidermal growth factor 2 receptors—have limited treatment options. Fewer than 30% of women with metastatic TNBC survive five years after their diagnosis, with a mortality rate within three months after a recurrence of 75%. Although TNBCs show a higher response to platinum therapy compared to other breast cancers, drug resistance remains a major obstacle; thus, platinum drugs with novel mechanisms are urgently needed. Arsenoplatins (APs) represent a novel class of anticancer agents designed to contain the pharmacophores of the two FDA approved drugs cisplatin and arsenic trioxide (As_2_O_3_) as one molecular entity. Here, we present the syntheses, crystal structures, DFT calculations, and antiproliferative activity of iodide analogs of **AP-1** and **AP-2**, i.e., **AP-5** and **AP-4**, respectively. Antiproliferative studies in TNBC cell lines reveal that all AP family members are more potent than cisplatin and As_2_O_3_ alone. DFT calculations demonstrate there is a low energy barrier for hydrolysis of the platinum-halide bonds in arsenoplatins, possibly contributing to their higher cytotoxicities compared to cisplatin.

## 1. Introduction

Between 15–20% of all breast cancer cases are classified as triple negative breast cancers (TNBCs): Patients in this category have the highest rate of metastatic disease and the poorest prognosis [1,2]. TNBC cells do not express well-established therapeutic targets including the estrogen and progesterone (ER and PR) receptors or human epidermal growth factor receptor 2 (HER2) [3]. Both the heterogeneity of the disease and the absence of well-established drug targets creates a challenge for the development of targeted therapy [4]. While neoadjuvant systematic treatment is the standard of care in the treatment of TNBC [5], median 5-year survival rates remain low. Therefore, there has been intense interest in characterizing mutations that may support the highly metastatic phenotype of TNBCs and employing this knowledge to develop better combination therapies. TNBCs with germline BRCA1/2 mutations are detected in 10–20% of patients with early-stage TNBC [6]. These cancers have DNA repair defects and are sensitive to DNA-damaging therapeutics such as platinum drugs [7]. Hence, platinum-based drugs emerged as a promising treatment option [8]. Phase 2 clinical studies with cisplatin against BRCA1 and BRCA2 mutated TNBCs have shown potential benefits [6]. Randomized clinical trials that include the addition of carboplatin to neoadjuvant taxane/anthracycline regimens improved pathologic complete response (pCR) in early-stage TNBC [9], and patients demonstrated an excellent 3-year recurrence-free survival rate of 90%. Additionally, a recent case study has shown success in using cisplatin monotherapy on a patient with heavily pretreated TNBC [6]. Currently, based on clinicaltrials.gov, there are 149 clinical trials with carboplatin, 63 clinical trials with cisplatin, and 7 trials with oxaliplatin against TNBCs. Although chemotherapy followed by surgery and radiotherapy is the main treatment option for TNBC patients, most experience disease progression [3]. Therefore, new metallodrugs with different mechanisms of action are urgently needed for the treatment of TNBCs, as has been highlighted elsewhere [10,11].

Another FDA approved anticancer drug, arsenic trioxide (ATO), has shown promise against TNBC [12]. The recent study by Teng and co-authors [13] has shown that the combination of FEN1 (Flap Endonuclease 1) inhibitors and low doses of ATO could be a promising therapeutic approach for TNBC patients. Recent studies have also shown significant anti-angiogenic activity for ATO in many cancers [14], including TNBC [15]. The discovery that the promyelocytic leukemia protein (PML) drives metastasis in TNBCs accelerated the urgency of finding a way to deliver ATO to TNBCs, as PML is targeted by ATO [16,17].

Inspired by the first nanoparticulate formulation of cisplatin and arsenous acid (platinum-arsenic nanobins) [18,19], we designed small molecular compounds that combine the pharmacophores of cisplatin and arsenous acid—arsenoplatins [19,20,21]. Arsenoplatins (APs) contain a robust Pt-As(OH)_2_ core held together by two chelate rings. Here, platinum(II) adopts its usual square planar geometry, but arsenic(III) possesses an unusual five-coordinated, trigonal bipyramidal geometry, with arsenic acting simultaneously as a Lewis acid and a Lewis base. Crystal structures of arsenoplatin-1 (**AP-1**), arsenoplatin-2 (**AP-2**), and an **AP-1** thiocyanate-derivate (**AP-3**), are the only known AP structures [20]. **AP-1**, the first member of the AP family, exhibits broad anticancer activity. In the one-dose NCI-60 screen, it was more potent than arsenic trioxide in all nine tumor types tested and more potent than cisplatin in breast, leukemia, CNS, and colon cancers and comparable to cisplatin in ovarian and renal cancers. Among all types of cancer tested, **AP-1** was the most potent against breast cancers and leukemia [21].

Messori and co-authors recently synthesized iodide analogs of cisplatin and oxaliplatin and compared their antiproliferative activities and interactions with DNA to those of the FDA approved drugs [22,23,24]. While earlier evaluation of an iodide analog of cisplatin by Cleare and Hoeshele suggested low activity [25], the expanded reexamination by Marzo et al. [23] compared the antiproliferative activity of cisplatin and its iodide analog complexes in several different human cancer cell lines: PANC-1 (pancreatic cancer), IGROV-1 (ovarian cancer), A549 (lung cancer), HT29 and HCT116-S (cisplatin sensitive) and HCT116-R (cisplatin resistant) colon cancer cell lines. In all investigated cancer cell lines except HCT116-S, the iodide analog was more potent than cisplatin (1.4–5.3 times). The iodide analog was taken up by cancer cells in greater amounts compared to cisplatin due to the greater lipophilicity of the iodide analog (logP values of −0.13 compared to logP values of cisplatin being −2.4). While the authors did not find a direct correlation between platinum uptake and cytotoxic activity, they found similar, albeit less efficient, DNA platination with the iodide analog [23].

With these results in mind, we synthesized iodide analogs of **AP-1** and **AP-2**, in this work, **AP-5**, and **AP-4**, and evaluated the potency of novel compounds against three TNBC cell lines.

## 2. Results and Discussion

### 2.1. Syntheses and X-ray Structures

**Scheme 1:**
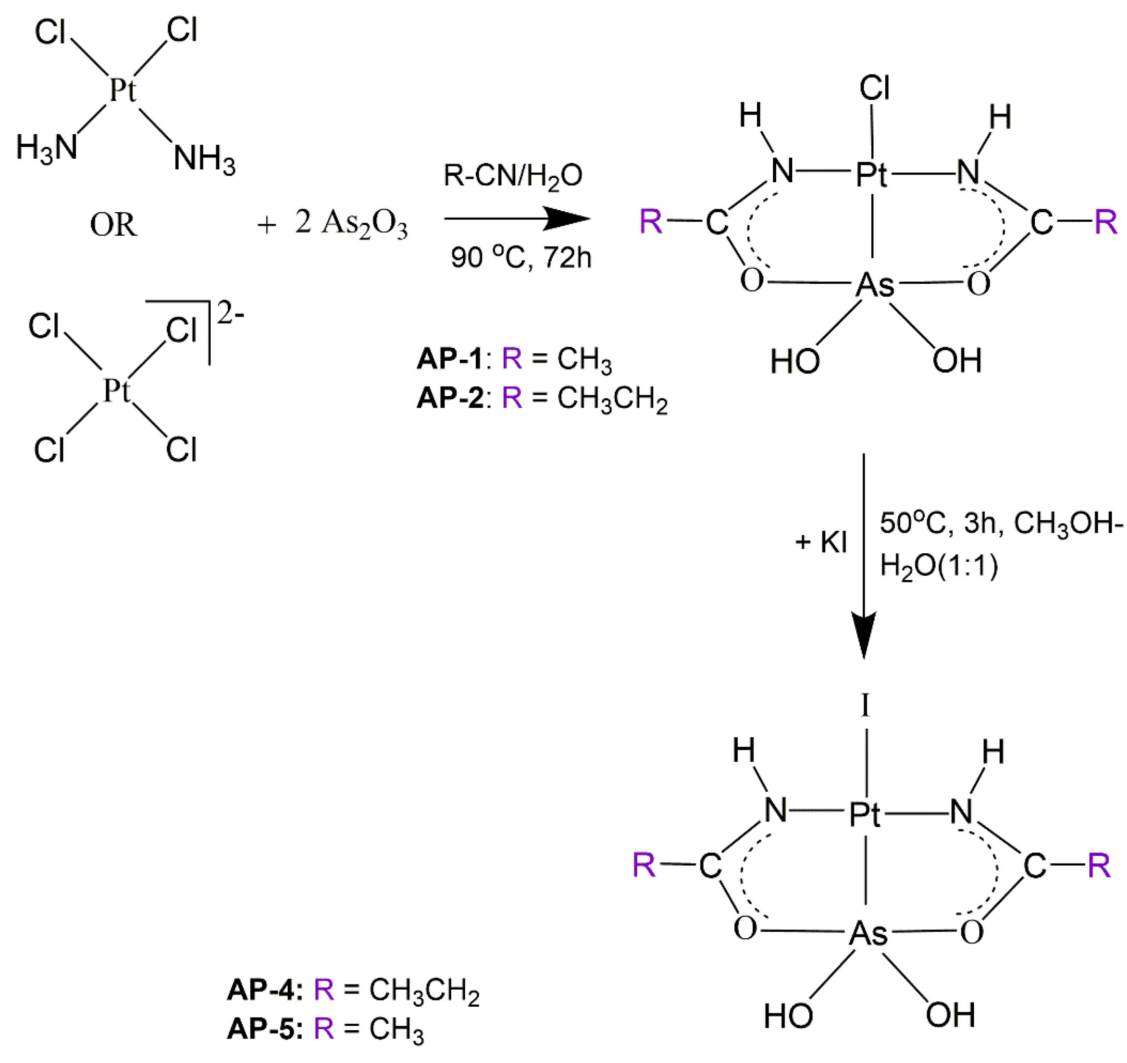
Syntheses of arsenoplatins.

Arsenoplatins **AP-5** and **AP-4** are synthesized from the previously reported **AP-1** and **AP-2** [20] by adding equimolar aqueous potassium iodide to the solutions of **AP-1** and **AP-2** in methanol and subsequent heating of the mixture at 50 °C for three hours, Scheme 1.

The presence of a strong sigma donor such as an arsenic atom in the *trans* position to chloride makes the chloride substitution reaction faster than cisplatin’s chloride substitution reactions [26]. The fast substitution of chloride by iodide, visible through an immediate change of the colorless solution to yellow, is facilitated by the *trans* effect of the arsenic moiety, and this is in agreement with our previous results [20].

**AP-4** and **AP-5** are freely soluble in DMSO and CH_3_OH. DMSO stock solutions of arsenoplatin compounds, like other drug candidates, were diluted into appropriate media for use in biological assays, including the NCI-60 screen [21]. ^1^H-NMR chemical shifts for **AP-4** and **AP-5** dissolved in DMSO-*d_6_* do not change over time, and no new peaks were observed (Figures S1 and S2), consistent with the idea that the Pt–Cl and Pt–I bonds in AP complexes do not undergo solvolysis in DMSO under these conditions. UV/Vis spectra of **AP-4** and **AP-5** in PBS solutions at pH = 7.4 or DMEM show no changes after one hour at 37 °C (Figures S3 and S4, See Supplementary Materials). Thus, the solution chemistry of these new congeners is similar to that of **AP-1** [21].

**Figure 1:**
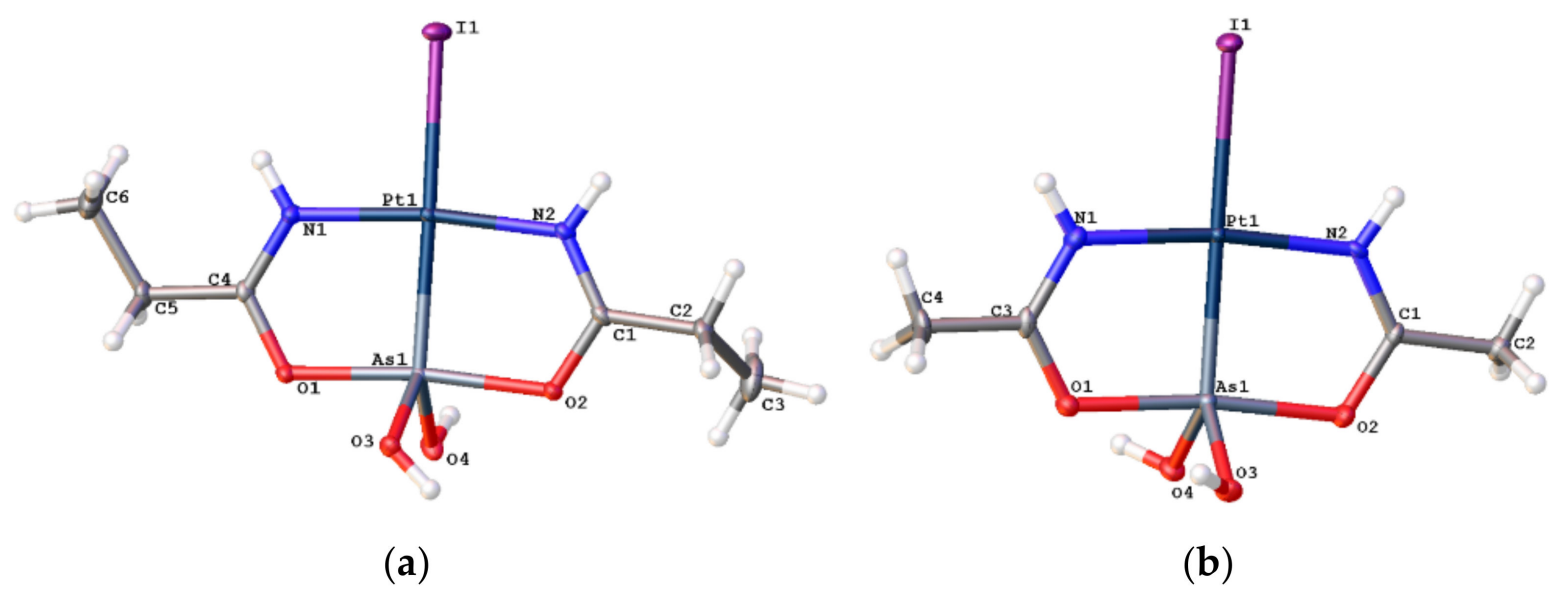
Thermal ellipsoid plots of **AP-4** (**a**) and **AP-5** (**b**) generated using Olex2. The plots are drawn at a 50% probability level. Water molecules in the crystal lattice of **AP-5** are omitted for clarity. Selected bond lengths and angles for **AP-4** and **AP-5** are given in Tables S1 and S2.

The solid-state structures of **AP-5** and **AP-4** were established using single crystal X-ray diffraction analysis (Figure 1). The Pt–I bonds in **AP-5** and **AP-4** are 2.6239(4) Å and 2.6143(3) Å (Table S1), comparable with the Pt-I bonds in [Pt(DACH)I_2_] (DACH = 1*R*,2*R*)-cyclohexane-1,2-diamine) of 2.60485 Å and 2.60690 Å [27] and other Pt(II)–I complexes [28,29,30].

The Pt–As core is robust and preserved during the substitution reaction of chloride ions in **AP-1** and **AP-2**. First, we note that the Pt–As bond lengths in **AP-5** and **AP-4** are 2.2834(7) and 2.2843(4) Å, respectively, ca. 0.01 Å longer than those seen in the respective chloride complexes (**AP-1** 2.2732(3) Å and **AP-2** 2.2687(4) Å), consistent with the stronger *trans* influence of an iodide relative to a chloride ligand [20].

Putting these Pt–As bond distances in a broader context, a CCDC search of 183 structures reveals Pt(II)–As bond lengths range from 2.2671 to 2.665 Å. The Pt–As bond lengths in **AP-5** and **AP-4** are 2.2834(7) Å and 2.2843(4) Å, respectively, and are among the very shortest Pt–As bond lengths reported in the literature [31]. The strength of this Pt–As bond and its kinetic stability [20] arise in part from the basicity of the As(III) lone pair and its ability to establish extensive overlap with Pt(II) d orbital.

The short Pt–As bond distance is also likely stabilized by the small bite distance of the bridging amidate ligand, which leads to two five-membered chelate rings. The N1–C4 and O1–C4 bond lengths in **AP-4** are 1.301(4) Å and 1.304(4) Å, and N2–C1 and O2–C1 bond lengths are 1.305(4) Å and 1.307(4) Å, respectively. In comparison, the N–C distance in acetamide is 1.38 Å and the C–O distance is 1.22 Å [32]. Thus, relative to a protonated amide, the N–C distances are ca. 0.08 Å shorter and the C–O distances are ca. 0.08 Å longer. This is consistent with a high degree of delocalization of the negative charge across the N–C–O bonds in an amidate ligands that bridge a Pt(II) and As(III) moiety. In **AP-5**, N1–C3 and O1–C3 bond lengths are 1.324(9) Å and 1.286(7) Å, and N2–C1 and O2–C1 are 1.296(7) and 1.300(8) Å, respectively; however, these distances are nonetheless consistent with a bridging amidate ligand. Combined, these features of the APs keep the platinum and arsenic pharmacophores together and contribute to the stability of the Pt-As cores of arsenoplatins in aqueous solutions.

### 2.2. Infrared Spectroscopy

**Figure 2:**
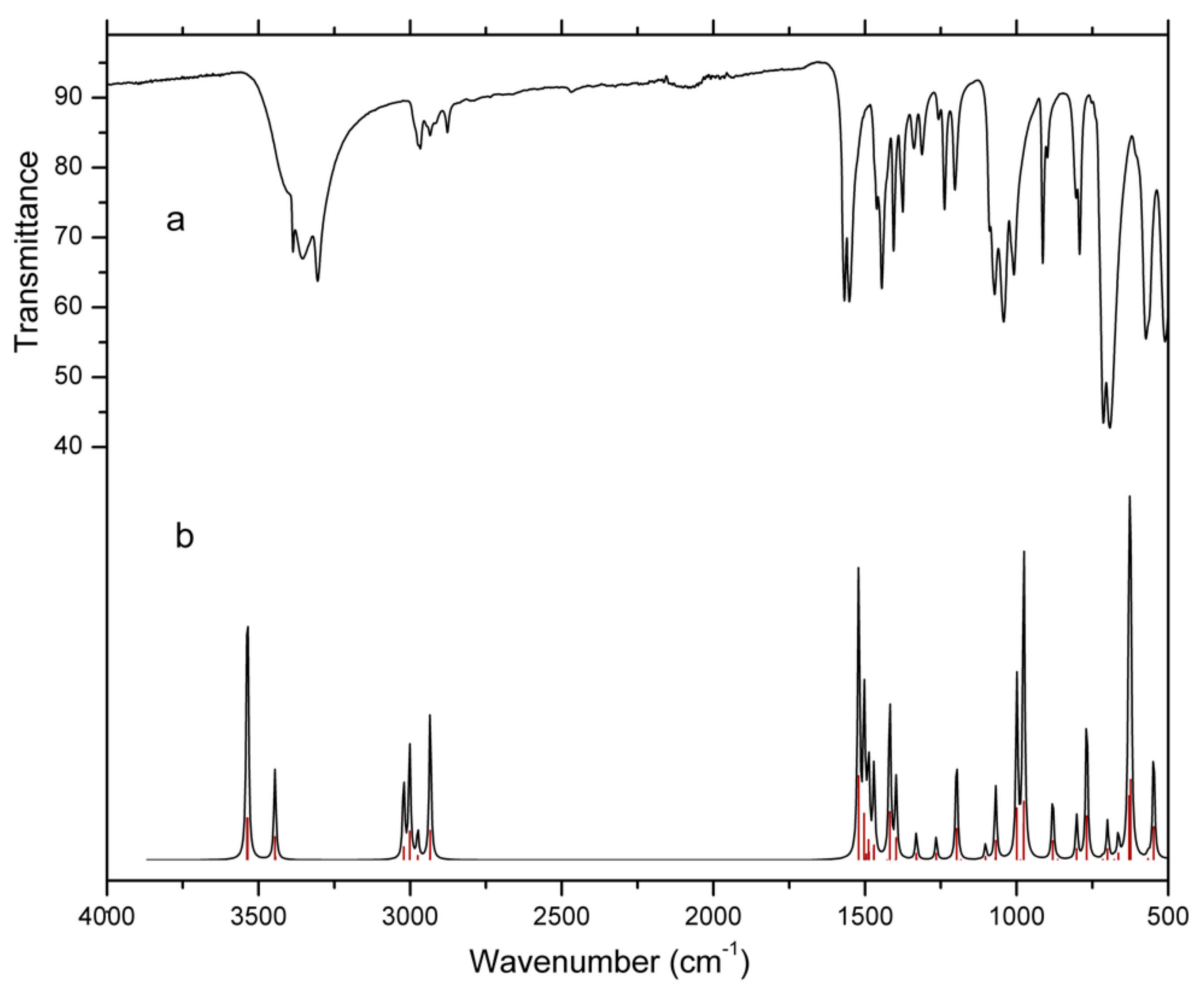
Experimental (**a**) and theoretical (**b**) FT-IR spectrum of **AP-4**. The red lines in the calculated spectrum denote a scaled frequency and infrared intensity of each vibration.

In the experimental FT-IR spectra of **AP-4** (Figure 2) and **AP-5** (Figure S5), the most intense bands have frequencies at around 700 cm^−1^. In **AP-4**, these strong vibrations are at 713 cm^−1^ and 693 cm^−1^. Based on the DFT normal mode analysis, these bands most likely arise from γO3–H + γO4–H + ν_asym_As–O3 (713 cm^−1^) and νAs–Pt + ν_sym_As–O3 + ν_sym_As–O4 + ν_asym_As–O4 (693 cm^−1^) vibrations, respectively (Table S3). The most intense band in the FT-IR spectrum of **AP-5** is at 712 cm^−1^. In the FT-IR spectrum of an aqueous solution of ATO (i.e., arsenous acid), As–O antisymmetric and symmetric stretches were observed at 800 cm^−1^ and 750 cm^−1^ [33]. Upon complexation, a significant shift of these bands in the FT-IR spectra of **AP-4** and **AP-5** toward lower frequencies is observed. Thus, the presence of this strong vibration at around 700 cm^−1^ can be used to confirm that the As(OH)_2_ moiety is bound directly to platinum(II). APs are still the only compounds to contain an arsenous acid moiety coordinated to the platinum(II) center.

The calculated vibrational frequencies are higher than the experimentally observed frequencies for most of the modes because the experimental value is an anharmonic frequency whereas the calculated value is a harmonic frequency. The uniform scaling procedure is applied to eliminate errors in the harmonic wavenumber output (in this work, 0.968) [34,35]. The second cause for some discrepancy between the calculated and experimentally observed vibrational frequencies is the environment of the molecules.

Bands at 3356 cm^−1^ and 3386 cm^−1^ in the experimental spectrum of **AP-4** are assigned to asymmetric and symmetric stretching vibrations of the O3−H and O4−H groups. These assignments are supported by scaled theoretical values of 3536 cm^−1^ and 3538 cm^−1^ (B3LYP mode nos. 80 and 81), respectively (Table S3). The stretching vibrations of N1−H and N2−H bonds at 3304 cm^−1^ correlate with the calculated value of 3446 cm^−1^ (B3LYP mode 79). The bands at 2877 cm^−1^, 2934 cm^−1^, and 2966 cm^−1^ in the FT-IR spectrum are due to asymmetric and symmetric C−H stretching vibrations of CH_3_ and CH_2_ groups and correlate well with the calculated values of 2934 cm^−1^, 2935 cm^−1^, and 3002 cm^−1^, respectively. The sharp bands at 1445 cm^−1^ and 1567 cm^−1^ are assigned to the stretching vibrations of C1−O2, C4−O1, and C1−N2, C4−N1 groups of **AP-4**, respectively.

The bands observed in the 1008 cm^−1^–1405 cm^−1^ region are caused by in-plane bending vibrations of N−H and O−H groups and correlate with the calculated values 976 cm^−1^–1398 cm^−1^ (B3LYP mode nos. 43, 45, 51, and 56). The bands observed at 713 cm^−1^ and 792 cm^−1^ in the FT-IR spectrum are assigned to the out-of-plane bending modes of these groups with counterparts calculated at 629 cm^−1^ and 769 cm^−1^ by the B3LYP method. As mentioned above, the band observed at 693 cm^−1^ has contributions from the As−Pt stretching and symmetric As−O3 and As−O4 stretching modes and agrees with the calculated 625 cm^−1^. The characteristic As−Pt group stretching vibrations are at 372 cm^−1^. Bending group vibrations likely account for the 195 cm^−1^ and 240 cm^−1^ bands in the low-frequency region of the calculated spectra of **AP-4** (not visible in the experimental FT-IR spectrum).

The experimental FT-IR spectrum of **AP-5** is similar to the FT-IR spectrum of **AP-4**; however, **AP-5** crystalizes with two water molecules. The symmetric and asymmetric O−H vibrations of water molecules are visible at 3553 cm^−1^ and 3446 cm^−1^ (Figure S5).

### 2.3. Cytotoxic Activity

Based on our previous NCI-60 screen of **AP-1**, which showed **AP-1** had the highest potency against breast cancers [21], we selected three TNBC cell lines, namely MDA-MB-231, MDA-MB-468, and MDA-MB-453, for further study. MDA-MB-468 is a Basal A (BL1), and MDA-MB-231 is a Basal B (BL2) subtype [36]. “Basal-like” are the most common subtypes of TNBCs, and patients with these types of cancer have the poorest clinical outcomes [37]. MDA-MB-453 is a luminal androgen receptor-positive TNBC cell line [36], although there is debate [38,39] as to whether this cell line should be classified as HER2-amplified or a TNBC [40]. Selected cancer cells are free of BRCA1 mutations [41,42]. We also included the non-tumorigenic cell line MCF-12A in this study. The MCF-12A cells are spontaneously immortalized cells derived from a women’s benign breast tissue with fibrocystic disease [43]. The MDA-MB-468 cell line is derived from an African American woman, and the MDA-MB-231 and MDA-MB-453 lines are derived from European American women. Our results showed that the MDA-MB-468 cell line has greater sensitivity to APs compared to cell lines obtained from European American women (MDA-MB-231 and MDA-MB-453), Table 1.

**Figure 3:**
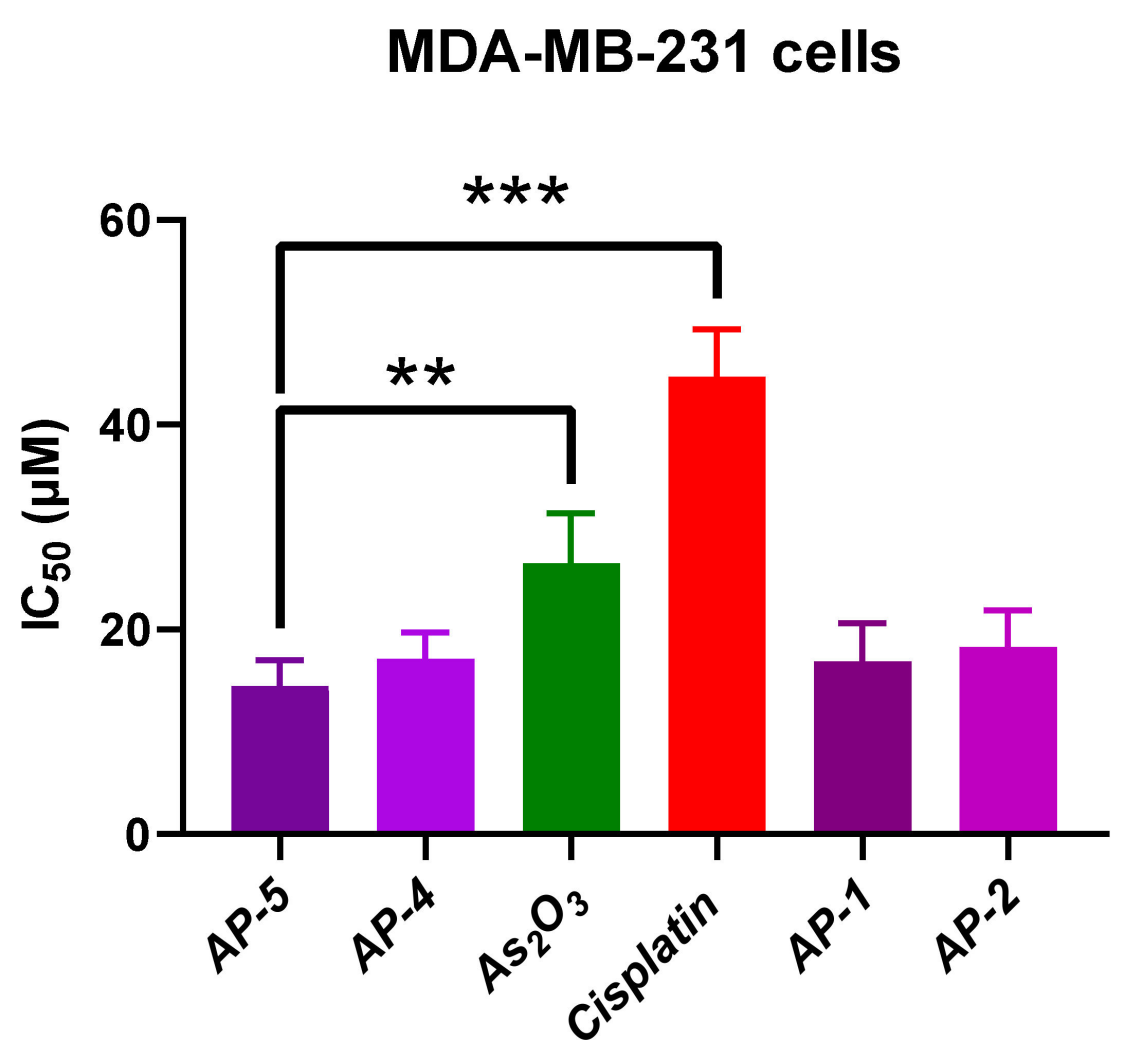
Cytotoxic activity of APs, cisplatin, and As_2_O_3_ against TNBC MDA-MB-321 cell line (72 h, MTS assay). The IC_50_ values were obtained from at least three independent experiments. There is a statistically significant difference in the cytotoxic activity of novel **AP-5**, cisplatin, and As_2_O_3_. Statistical differences were determined by one-way ANOVA and followed by unpaired *t*-test (** *p* ≤ 0.01, *** *p* ≤ 0.001).

Based on the IC_50_ values from cytotoxicity assays, APs are more potent than cisplatin and/or ATO in all investigated TNBC cell lines, although there is a variation among different TNBC cell lines. The significance given in Table 1 is related to the cytotoxicity of **AP-5** compared to all other compounds. There is a significant difference in the cytotoxicity of **AP-5** compared to the cytotoxicities of ATO and cisplatin in the MDA-MB-231 TNBC cell line, as shown in Figure 3. There is no statistically significant difference in the cytotoxic activity between APs with platinum bonded chloride/iodide; for example, **AP-1,** and its iodide analog, **AP-5**. This agrees well with the results of the DFT calculations for the energetics of the hydrolysis processes for **AP-1** and **AP-5**. Both processes have almost the same activation barriers, 7.4 and 8.7 kcal/mol, respectively (see Section 2.4 Path I, Figure 5). After the hydrolysis is complete, the aqua complexes of **AP-1** and **AP-5** have identical structures, so their behaviors in the cellular milieu are expected to be the same.

The toxicity of APs toward non-tumorigenic MCF-12A is comparable to that of cisplatin. **AP-4** is significantly less toxic towards this cell line compared to **AP-5**. The toxicities of APs are comparable to the toxicity of ATO, except for **AP-5,** which is significantly more toxic than ATO. Although ATO showed potency against many solid tumors in vitro [44], ATO suffers from rapid renal clearance in vivo [45,46,47]; therefore, an intense search to develop methods to deliver ATO to solid tumors is ongoing [12,47,48,49]. APs may be a simple solution to this problem, as they can deliver platinum and arsenic pharmacophores simultaneously. To decrease toxicity toward normal cells or animals, different delivery methods for APs are under consideration.

### 2.4. DFT Calculations

Density functional theory (DFT) played a pivotal role in recognizing the key activation step in the anticancer mechanisms of cisplatin [50,51] and other platinum drugs [52,53]. All DFT calculations in this work were performed using the B3LYP hybrid exchange-correlation functional [54,55] implemented in Gaussian09 code [56]. B3LYP is widely used functional for geometry optimization of platinum compounds and for the subsequential electronic energy calculations [57,58]. The bond lengths obtained by X-ray diffraction (XRD) were compared with the bond lengths obtained at the theoretical level (Table S4). The calculated bond lengths are slightly longer than the experimental values, as was previously observed for the [Pt(DACH)I_2_] complex [27], **AP-1** [57], and the AP-SCN complex **AP-3** [58]. The largest discrepancies between experimental and theoretical bond lengths are for the Pt–As bond (in **AP-4** 0.0937 Å in vacuum and 0.0875 Å in water, and in **AP-5** 0.096 Å in vacuum and 0.0888 Å in water). These slight discrepancies between calculated and experimental bond lengths may be attributed to the fact that the XRD values correspond to solid state measurements whereas the DFT calculations were performed using isolated molecules in vacuum or water simulated environments. The comparison between experimental and calculated bond angles (°) is presented in Table S5.

#### The Energetics of the Hydrolysis Reaction

**Figure 4:**
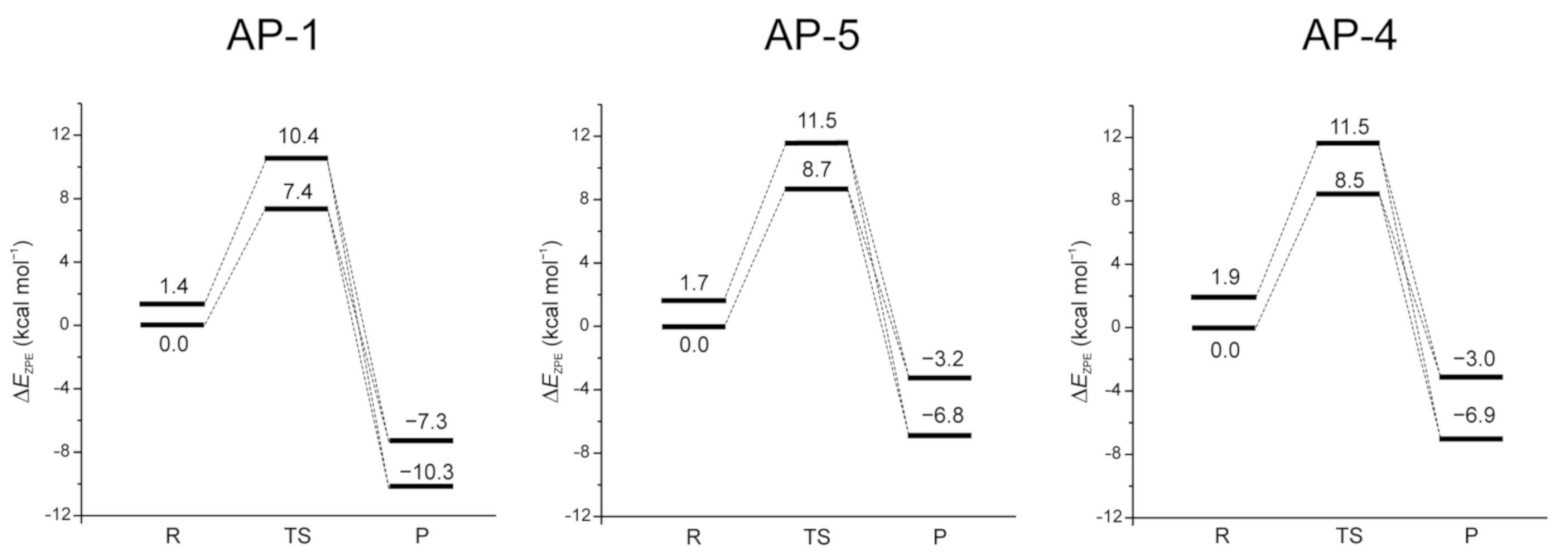
The calculated B3LYP zero-point energy profiles for the aquation processes of **AP-1**, **AP-5**, and **AP-4** with two explicit water molecules included in the calculations. Energies are in kcal/mol and are relative to the most stable reactant (R = reactant, TS = transition state, P = product).

Inspired by previous investigations of the hydrolysis processes of cisplatin [59,60] and other platinum drugs [61,62], as well as **AP-1** and **AP-3** [57,58], we explored the energetics of the hydrolysis processes of the novel **AP-4** and **AP-5** complexes. DFT calculations with two additional explicit water molecules included in the calculations, in addition to the nucleophilic water molecule, resulted in the exothermic aquation and hydrolysis reactions of **AP-4** and **AP-5** and for the previously synthesized compound **AP-1** [20], (Figure 4). Explicit water molecules were included in the calculations to address the criticism that using only one water molecule neglects water-water interactions in the hydrolysis process [53,63]. Additional explicit (nonbonding) water molecules are included to incorporate hydrogen bonding between water molecules and between water and leaving Cl^−^ and I^−^ ions. Overall, this improves solvation energies [62,64] and provides a more accurate description of the stabilization of the leaving ligand [64].

**Figure 5:**
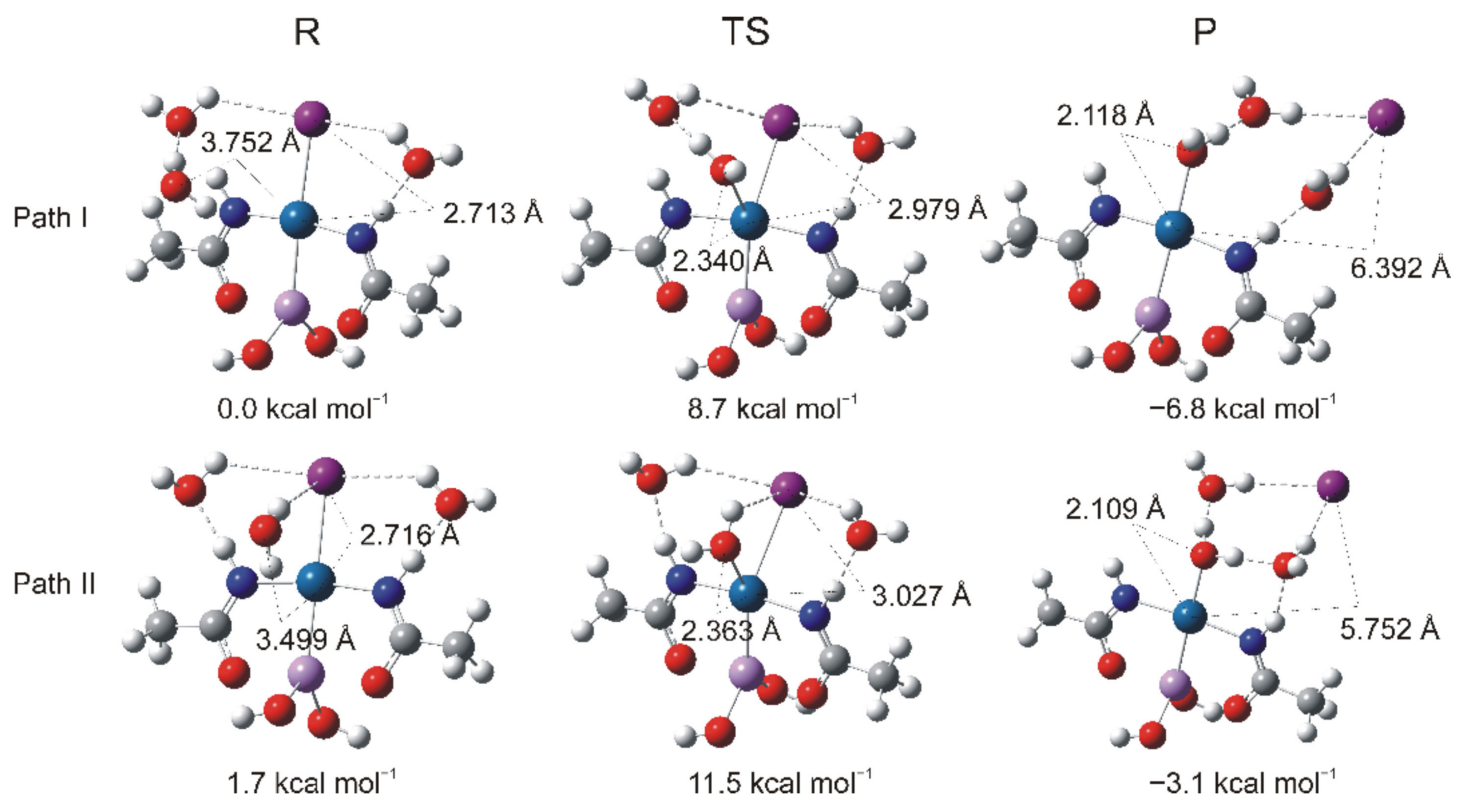
Geometries of species involved in the hydrolysis of **AP-5** with two explicit water molecules included in the calculation. All energies are relative to the most stable reactant and are given in kcal/mol. There is one additional reactant geometry with 1.7 kcal/mol (Path II). Two different microstates are due to the orientation of water molecules and H-bonds formed with **AP-5** (R = reactant, TS = transition state, P = product). Color code: Pt—blue, I—purple, As—pink, N—dark blue, O—red, C—gray, H—white.

From the optimized geometries and X-ray crystal structures, it is visible that APs have nearly planar coordination around the platinum center. Several hydrogen bond donors and acceptors are present, indicating that the first solvation sphere includes many water molecules but only a few are close to the platinum center. Close observation of the optimized structure revealed organization of water molecules near deprotonated amide rings. These water molecules can serve as reacting water molecules in the hydrolysis reaction (substitution of Cl^−^ ion in **AP-1** by a water molecule) or as H-bond acceptors/donors that may “lock” the position of an approaching water molecule. To better compare these possible reaction paths, we defined several systems with a different number of explicit water molecules combined with the conductor-like polarizable continuum model (C-PCM) method [65,66] to define the energy relationship in hydration of APs as best as possible (Figure 5 and Figures S6 and S7). The arsenic atom in the *trans* position to the platinum-chloride/iodide bond facilitates the hydrolysis process through its strong *trans* effect [67,68]. The substitution of the Cl^−^ ion in **AP-1** by a water molecule is slightly more exothermic compared to the process of substitution of the I^−^ ions by water in **AP-4** and **AP-5**, which may be explained by the stronger interactions of the smaller chloride ion with water molecules compared to the interactions of the significantly larger iodide ion with water molecules. The slightly exothermic aquation process supports the experimental observation that the substitution of the chloride ion with the iodide ion is fast, and in the case of substitution of the chloride ion by SCN^−^, instantaneous at room temperature in aqueous solution [20]. The fully optimized structures for the species involved in hydrolysis with two additional explicit water molecules are presented in Figure 5.

In the system with two explicit water molecules in addition to the nucleophilic water molecule, we find numerous reaction microstates differing by only a few kcal/mol. Examination of those geometries reveals two characteristic reactive states and paths (Figure 5). In Path I, the incoming water molecule forms a hydrogen bond with the water molecule above the NH group. This positions the water above the Pt reaction center and brings it to an almost ideal position for interaction between a lone pair of the oxygen atom and the unoccupied d-orbital on the Pt atom. In Path II, the incoming water molecule forms a weak O–H^…^X hydrogen bond with the leaving X ion (Cl^−^or I^−^), which positions it above the Pt center. However, in this state, neither of the oxygen lone pairs are well-oriented for the interaction with the d-orbital on the Pt atom. Consequently, the reacting complex for **AP-5, AP-1**, and **AP-4** has 1.7 kcal/mol, 1.4 kcal/mol, and 1.9 kcal/mol higher energy than the reacting complex in Path I (Figure 5 and Figures S6 and S7). Two reactant geometries for **AP-5** have a similar Pt–I bond length, 2.713 Å, and 2.716 Å, respectively (Figure 5). The fully optimized structure of **AP-5** is in good agreement with the crystallographic data, with the Pt–I bond length distances within 0.0891 Å and 0.0893 Å (Table S4).

In Path I, the entering water molecule is 3.752 Å from the platinum center. That distance reduces to 2.340 Å in the transition state. In the transition state, the Pt–I bond increases from 2.713 Å to 2.979 Å. The bond-breaking and bond-forming occur simultaneously within a trigonal bipyramidal transition state at the Pt(II) atom and are consistent with a concerted S_N_2 reaction process, as was also seen in the hydrolyses of the orally active anticancer drug ZD0473 (picoplatin) [61] and of cisplatin [50]. The activation energy is 8.7 kcal/mol. In the product, the iodide ion is completely substituted by a water molecule. The Pt–OH_2_ bond length is 2.118 Å and is comparable with other calculated Pt(II)–OH_2_ bonds [68,69]. The energy change for the reaction represented in Path I is −6.8 kcal/mol.

In Path II, the entering water molecule is 3.499 Å from the platinum center, and that distance reduces to 2.363 Å in the transition state. In the transition state, the Pt–I bond increases from 2.716 Å to 3.027 Å. The activation energy is 11.5 kcal/mol. In the product, the iodide ion is completely substituted by a water molecule with the Pt–O bond of 2.109 Å. The energy change for the reaction represented in Path II is −3.1 kcal/mol.

Our results agree with the DFT (CAM-B3LYP) calculation results performed by Re and coauthors for the substitution reaction of the chloride in the **AP-1** complex with the thiocyanate ion (**AP-3**). The authors found that the substitution reaction is exergonic [58], which explains the rapid substitution of Cl^−^ with SCN^−^ ion in an aqueous solution at room temperature [20].

Results of the DFT calculations without explicit water molecules, as well as with one explicit water molecule for the hydrolysis of **AP-1**, **AP-4**, and **AP-5,** have shown large differences in activation and reaction energies (Figure S7). To more realistically mimic reactions in aqueous solution, we believe that at least three water molecules should be included in the simulation.

In the cytoplasm, the hydrolysis reaction is the first step in the activation of platinum drugs [50,64,70]. Russo and co-authors compared the energy barriers of **AP-1′s** and cisplatin’s hydrolysis processes and found that the hydrolysis of **AP-1** is more favorable [57]. Depending on the basis set used for DFT calculations, the activation barriers for hydrolysis of cisplatin are in the 25 kcal/mol–30 kcal/mol range [51,57]. The higher cytotoxic activities of APs compared to cisplatin could be due to the lower energy barrier required for the aquation process. The process of hydrolysis in arsenoplatins is facilitated by the presence of the arsenic atom and its strong *trans* effect. Natural bond orbital (NBO) analysis shows an almost neutral charge on the Pt of **AP-5** (−0.05198|e|) and of **AP-4** (−0.05235|e|) in water (Table S6). The negative charges on iodine in **AP-5** and **AP-4** are almost the same, −0.34452|e| and −0.34451|e|, respectively. Russo and co-authors performed NBO analysis for **AP-1** and cisplatin and obtained an almost neutral charge on platinum in **AP-1** as of 0.043|e| and 0.214|e| in cisplatin. The higher positive charge on platinum in cisplatin is consistent with the idea that release of the chloride ion from platinum is more difficult in cisplatin compared to **AP-1**. The authors have also shown that guanine binding is energetically preferred for **AP-1** compared to cisplatin [57].

The DFT calculations performed support the idea that the aquation process in APs is exothermic. We postulate that this contributes to the higher cytotoxicity of arsenoplatins against TNBC (Table 1) and other cancer cell lines compared to cisplatin [21].

Our previous results using ICP-MS measurements confirmed the presence of both platinum and arsenic in DNA samples isolated after the treatment of TNBC MDA-MB-231 cells with **AP-1**, which indicates that **AP-1** binds to DNA [21]. Based on this earlier work, we expect the iodide analog **AP-5** to bind DNA similarly. After water substitution for the chloride/iodide ion, the aqua complexes of **AP-1** and **AP-5** will have the same structure, so we anticipate similar behavior in the biological medium. Although our earlier data clearly indicates that **AP-1** binds DNA, this attribute may not be essential to its mechanism of action. Intriguingly, in the NCI-60 screen, a higher Pearson correlation coefficient (PCC) is obtained when the response of **AP-1** is compared to the response of ATO (PCC = 0.78) than when the response of **AP-1** is compared to the response of cisplatin (PCC = 0.67) [21]. Future studies will address the extent to which the As(III) and Pt(II) moieties are involved in the anticancer activity of arsenoplatins.

## 3. Materials and Methods

### 3.1. General Experimental Considerations

The arsenoplatins **AP-1** and **AP-2** were prepared according to their reported procedures [20]. Commercially available chemicals were purchased through Sigma-Aldrich (Darmstad, Germany). Combustion analysis was performed at Northwestern University using the Elementar Vario-El Cube instrument. Ambient temperature NMR spectra were recorded on the Bruker Advance III 500 MHz system is equipped with a DCH CryoProbe. The FT-IR spectra were recorded using Nicolet iS5 Infrared Spectrometer (Thermo Scientific). The electron absorption spectra were recorded using a PerkinElmer Lambda 650 UV/VIS Spectrometer.

### 3.2. Syntheses of Arsenoplatin Compounds

Arsenoplatin-4, **AP-4**: 1.25 × 10^−4^ mol (60.3 mg) of **AP-2** was dissolved in 3 mL of CH_3_OH, and then an equimolar aqueous solution of KI was added. The reaction mixture was heated for 3 h at 50 °C. Crystals suitable for X-ray analysis are obtained after standing of the mother liquor for several days at room temperature. Yield 37.6 mg (52%). The purity of the novel arsenoplatin compound is confirmed by elemental analysis: Calculated for C_6_H_14_AsIN_2_O_4_Pt, 12.53% C, 2.46% H, 4.87% N, Found 12.60% C,1.99% H, 4.92% N. ^1^H-NMR (600 MHz, [D_6_]DMSO): 8.95 ppm (s, 2H-OH); 7.92 ppm (s, 2H-NH); 2.50 ppm (4H-CH_2_—signal overlaps with D5H DMSO, 1.05 ppm (s, 6H-CH_3_). UV/Vis of 1 × 10^−4^ M (PBS, pH = 7.4) solution: λ_1_ = 284 nm, ε_1_ = 4951 M^−1^cm^−1^, λ_2_ = 223 nm, ε_2_ = 19579 M^−1^cm^−1^. FT-IR (cm^−1^): 3386(m), 3356(m), 3304(s), 1567(s), 1555(s), 1445(s), 1405(m), 1073(m), 1042(s), 1008(s), 914(m), 792(m), 713(vs), 693(vs), 572(s), 509(s). NMR and UV/Vis spectra of **AP-4** are available in Figures S1 and S3.

Arsenoplatin-5, **AP-5**: 2.5 × 10^−4^ mol of AP-1 was dissolved in 3 mL of CH_3_OH, and then an equimolar aqueous solution of KI was added. The reaction mixture was heated for 3 h at 50 °C. Crystals suitable for X-ray analysis are obtained after standing of the mother liquor for several days at room temperature. Yield 88%. The purity of the novel arsenoplatin compound is confirmed by elemental analysis: **AP-5**, Calculated for C_4_H_10_AsIN_2_O_4_Pt·H_4_O_2_: 8.78% C, 1.85% H, 5.1% N, Found 8.93% C, 1.4% H, 5.16% N. ^1^H-NMR (600 MHz, *d*_6_-DMSO): 8.96 ppm (s, 2H-OH); 8.12 ppm (s, 2H-NH); 2.17 ppm (s, 6H-CH_3_). UV/Vis 1 × 10^−4^ M (PBS, pH = 7.4): λ_1_ = 284 nm, ε_1_ = 3868 M^−1^cm^−1^, λ_2_ = 225 nm, ε_2_ = 15867 M^−1^cm^−1^. FT-IR (cm^−1^): 3536(w), 3446(w), 3270(m), 1628(w), 1557(s), 1463(w), 1418(s), 1372(w), 1138(m), 1038(w), 962(m), 834(m), 717(vs), 630(m), 585(s). NMR and UV/Vis spectra of **AP-5** are available in Figures S2 and S4.

### 3.3. Crystallographic Measurements

The crystallographic measurements were performed using a Bruker Kappa APEX CCD (Madison, WI, USA) area detector diffractometer. Data collection: APEX2 V2017.3; Cell refinement: SAINT V8.34A; Data reduction: Bruker SAINT; programs used to solve structure: XS and XL (Sheldrick) [71] and Olex 2 [72]. Thermal parameters were refined anisotropically for all non-hydrogen atoms. Hydrogen atoms are at calculated positions, and methyl hydrogens were added via HFIX33. The **AP-5** crystal was found to have significant twinning, which was accounted for via a multiscan absorption correction. Solvent (water) was not masked and instead was refined with the structure, exhibiting rational disorder and hydrogen bonding. CCDC # 2092028 (**AP-5**) and CCDC # 2092029 (**AP-4**) contain the supplementary crystallographic data. These data can be obtained free of charge via https://www.ccdc.cam.ac.uk/structures/ (accessed on 24 June 2021) (or from the CCDC, 12 Union Road, Cambridge CB2 1EZ, UK; Fax: +44-1223-336033; E-mail: deposit@ccdc.cam.ac.uk).

Crystal Data for C_6_H_14_AsIN_2_O_4_Pt, **AP-4** (M = 575.10 g/mol): orthorhombic, space group *Pbca*, a = 14.3348 (14) Å, b = 9.6998 (9) Å, c = 18.6480 (18) Å, V = 2592.9 (4) Å^3^, Z = 8, T = 100 K, μ (Mo*Kα*) = 0.71073 Å, Dcalc = 2.946 g/cm^3^, 29719 reflections measured (2.2° ≤ 2θ ≤ 36.3°), 6273 unique (Rint = 0.050, Rsigma = 0.0448) which were used in all calculations. The final R1 was 0.030 (I > 2σ(I)) and wR2 was 0.061 (all data).

Crystal Data for C_4_H_10_AsIN_2_O_4_Pt·H_4_O_2_, **AP-5** (M = 583.08 g/mol): monoclinic, space group (*P 12_1_/m1*), a = 8.7720 (3) Å, b = 6.6429 (2) Å, c = 11.5977 (3) Å, α = 90°, β = 111.8840(10)°, γ = 90°, V = 627.12 (3) Å^3^, Z = 2, T = 101 K, μ (Mo *Kα*) = 0.71073 Å, Dcalc = 3.088 g/cm^3^, 2065 reflections measured (1.9° ≤ 2θ ≤ 30.5°), 2065 unique (Rint = 0.0578, Rsigma = 0.0500) which were used in all calculations. The final R1 was 0.0562 (I > 2σ(I)) and wR2 was 0.074 (all data).

### 3.4. Biological Studies

#### 3.4.1. Cell Lines and Cell Culture

Human breast epithelial non-tumorigenic MCF-12A cells and tumorigenic MDA-MB-231, MDA-MB-468, and MDA-MB-453 cells were obtained from ATCC. Authentication of cell lines was performed by short tandem repeat (STR) profiling at Northwestern NUseq Core Facility. The resulting autosomal STR profiles were compared and matched 100% to the ATCC database.

MCF-12A was cultured in Dulbecco’s modified Eagle’s medium (DMEM), and supplemented DMEM/F12 medium (GIBCO) was added with 5% horse serum (GIBCO), 0.02 μg/mL human epidermal growth factor (EGF, Sigma-Aldrich), 0.5 μg/mL hydrocortisone (Sigma-Aldrich), 0.1 μg/mL cholera toxin (Sigma-Aldrich), 10 μg/mL insulin (Sigma-Aldrich), and 1% penicillin-streptomycin (GIBCO). MDA-MB-231, MDA-MB-468, and MDA-MB-453 were cultured in Dulbecco’s modified Eagle’s medium (DMEM) and supplemented with 10% Fetal Bovine Serum (FBS, GIBCO) and 1% penicillin-streptomycin (GIBCO). Cells were grown at 37 °C in a humidified atmosphere of 5% of CO_2_.

#### 3.4.2. Cell Viability Assay

The 50% growth inhibition concentration (IC_50_) of arsenoplatins, cisplatin, and ATO (IC_50_ assay) was determined in breast cancer cell lines of MDA-MB-231, MDA-MB-453 and MDA-MB-468 by measuring the optical density using the CellTiter 96 Aqueous MTS assay according to the manufacturer’s instructions (Promega, Madison, WI, USA) as previously described [73]. Briefly, cells were plated in triplicate or sextuplicate in the wells of 96-well plates, and cell proliferation and viability were quantified. Cells were plated at the optimized density for each cell line in 96-well plates and kept in an incubator overnight at 37 °C in a humidified atmosphere of 5% CO_2_. The cells were treated with serial dilutions of APs and drugs in the growth media for about 72 h. The cells were then pulsed with 20 μL of a freshly prepared solution of MTS plus phenazine methosulfate. After incubation for up to 1 h at 37 °C, the absorbance at 490 nm was recorded with a BioTek Synergy 2 plate reader (BioTek, Winooski, VT, USA). The IC_50_ value for each compound was calculated using a non-linear regression model of four parameters variable slope using GraphPad Prism 9.1 software (GraphPad, San Diego, CA, USA). The IC_50_ values were obtained from at least three independent experiments.

### 3.5. DFT Calculations

All DFT calculations were performed by the Gaussian09 code [56], employing the B3LYP hybrid exchange–correlation functional [54,55]. For Pt and As atoms, the relativistic compact Stuttgart/Dresden effective core potential and associated split valence basis set [74] were used. The 6-311G(d,p) basis sets were used for Cl and I, and the standard 6-311++G(d,p) basis sets were used for the remaining atoms. The geometries for all species have been optimized in water by using the C-PCM method [65,66]. To verify the proper character of all stationary points (minimum or saddle point) and to compute the zero-point vibrational energy (ZPVE) corrections, the harmonic frequencies were computed and analyzed for all optimized geometries. The computed frequencies were scaled by 0.968 for assignments to the experimental IR spectra [35]. The assignments of the calculated wavenumbers were assisted by the animation option of GaussView 5.0 graphical interference [75] from Gaussian programs, which performs a visual presentation of the shape of vibrational modes. The natural bonding orbitals (NBOs) [76] analysis was performed to evaluate the NBO partial atomic charges on the atoms involved in the coordination of the metal ion for each species studied.

### 3.6. Statistical Analysis

Statistical analysis was performed using GraphPad Prism 9.1 software (GraphPad, San Diego, California, USA). All data are presented as mean +/− SD (standard deviation) from at least three independent measurements. Statistical differences were determined by one-way ANOVA with *p* < 0.05 considered statistically significant.

## 4. Conclusions

The results presented in this manuscript are part of our ongoing effort to explore the reactivity and cytotoxicity of arsenoplatins, a new class of dual pharmacophore anticancer agents. We focused our investigation on the evaluation of the cytotoxic activity of new arsenoplatin compounds, **AP-4** and **AP-5**, against TNBC cell lines. The solid-state structures of **AP-4** and **AP-5** were solved via single crystal X-ray analysis. Based on the results of antiproliferative studies, arsenoplatins are more potent than the FDA approved drugs cisplatin and arsenic trioxide in all investigated cancer cell lines. There is no significant difference in the cytotoxicity between arsenoplatins with chloride (**AP-1** and **AP-2**) or iodide (**AP-4** and **AP-5**) bound to platinum. The presence of the arsenic atom in the *trans* position to platinum facilitates the hydrolysis process, which is the first step in the activation of platinum drugs. DFT calculations with two explicit water molecules resulted in the exothermic hydrolysis process. The difference in energy of the hydrolysis processes of arsenoplatins compared to cisplatin have a direct effect on the anticancer activity and cytotoxicity of these compounds and could be one of the main reasons for the higher cytotoxicities of arsenoplatins compared to cisplatin. Whether the As(OH)_2_ moiety is involved in the anticancer activity more directly, besides by weakening the platinum-halide bond through the kinetic *trans* effect imposed by the arsenic atom, is yet to be determined.

## Figures and Tables

**Table 1:** Results of the cytotoxic activity of APs, ATO (as As(OH)_3_), and Cisplatin from at least three independent experiments (IC_50_ values in µM for 72 h treatment). The statistically significant difference is related to the cytotoxicity of novel **AP-5**.

	MDA-MB-231	MDA-MB-453	MDA-MB-468	MCF-12A
**AP-5**	14.5 ± 2.52	12.70 ± 2.52	2.11 ± 1.89	15.47 ± 2.87
**AP-4**	17.16 ± 2.55	13.23 ± 2.65	1.70 ± 0.76	25.02 ± 2.14 (**)
**AP-1**	16.94 ± 3.70	12.02 ± 1.34	1.88 ± 0.88	23.43 ± 6.62
**AP-2**	18.31 ± 3.59	13.75 ± 3.26	2.05 ± 0.87	23.84 ± 4.12
As(OH)_3_	26.49 ± 4.88 (**)	15.63 ± 1.70	3.90 ± 0.21 (**)	27.25 ± 3.46 (*)
Cisplatin	44.7 ± 4.63 (***)	20.87 ± 0.85 (**)	2.26 ± 0.33	19.42 ± 6.48

Statistical differences were determined by one-way ANOVA and followed by unpaired *t*-test. (* *p* ≤ 0.05, ** *p* ≤ 0.01, *** *p* ≤ 0.001).

## Data Availability

The data is available in the Supplementary Materials accompanying this article.

## Supplementary Materials

The following are available online, Tables S1 and S2: Selected bond lengths (Å) and angles (°) for **AP-4** and **AP-5**; Table S3: The most significant observed FT-IR and calculated frequencies with B3LYP method for **AP-4**; Table S4: Bond distances (Å) obtained at the theoretical level (in vacuum and water) and their experimental counterpart; Table S5: Comparison of bond angles (^o^) obtained by XRD and DFT (B3LYP) calculations in water and vacuum; Table S6: Natural bond orbital charge (e^−^) for **AP-1**, **AP-4,** and **AP-5**; Figures S1 and S2: ^1^H NMR spectra of **AP-4** and **AP-5**; Figures S3 and S4: UV/Vis spectra of **AP-4** and **AP-5**; Figure S5: FT-IR spectrum of **AP-5**, Figure S6: Geometries of species involved in the hydrolysis of **AP-5** with one or two water molecules included in the calculation; Figure S7: The calculated B3LYP ZPE energy profiles for the aquation process of **AP-1**, **AP-5**, and **AP-4** with one, two, and three water molecules included in the calculation.

## Sample Availability

The compounds described in this paper were not synthesized in sufficiently large amounts to be available for distribution at this time.
