# Supplementary material for: Iodide Analogs of Arsenoplatins—Potential Drug Candidates for Triple Negative Breast Cancers
Source: Molecules. 2021 Sep 6;26(17):5421. doi: 10.3390/molecules26175421 (PMC8434261; doi:10.3390/molecules26175421)
Supplement: Supplementary file 1 [file molecules-26-05421-s001.zip › molecules-1342904-supplementary.pdf]

Iodide Analogs of Arsenoplatins – Potential Drug Candidates for Triple Negative Breast Cancers

Đenana Miodragović[1,2], Wenan Qiang[2,3], Zohra Sattar Waxali[2], Željko Vitnik[4], Vesna Vitnik[4], Yi Yang[2], Annie Farrell[4], Mathew Martin[1], Justin Ren[2], and Thomas V. O'Halloran[2,6]

[1]Northeastern Illinois University, 5500 St. Louis Ave, Chicago 60625, USA

[2]Chemistry of Life Processes Institute, Northwestern University, 2170 Campus Drive Evanston, Illinois 60208 USA

[3]Department of Obstetrics and Gynecology, Feinberg School of Medicine, Northwestern University, 303 East Superior Street, Chicago, 2170 Campus Illinois 60611

[4]Institute of Chemistry, Technology, and Metallurgy, University of Belgrade, Njegoševa 12, Belgrade 11000, Serbia

[5]University of Illinois at Urbana Champaign, 102 N. Neil St. Champaign, Illinois 61820, USA

[6] Department of Chemistry and Department of Microbiology & Molecular Genetics Michigan State University, East Lansing, 567 Wilson Road, Michigan, 48824, USA

Correspondence: ohallor8@msu.edu; t-ohalloran@northwestern.edu; Tel.: 847-491-5060; Fax: 847-467-156

Supporting Information:

Tables

Table S1: Selected Bond Lengths (Å) for **AP-4** and **AP-5**.

Table S2: Selected Bond Angles (°) for **AP-4** and **AP-5**.

Table S3: The most significant observed FT-IR and calculated frequencies with B3LYP method for AP-4 [harmonic frequency (cm^{-1}), IR_{int} (K $mmol^{-1}$)].

Table S4: Bond distances (Å) obtained at the theoretical level (in vacuum and water) and their experimental counterpart (R^2 correlation coefficient between DFT and XRD data).

Table S5: Comparison of bond angles (°) obtained by XRD and DFT (B3LYP) calculations in water and vacuum.

Table S6: Natural bond orbital charge (e^-) for **AP-1**, **AP-4,** and **AP-5**.

Figures

Figure S1: ^1H NMR spectra of **AP-4** acquired at 500 MHz in DMSO-d_6.

Figure S2: ^1H NMR spectra of **AP-5** acquired at 500 MHz in DMSO-d_6.

Figure S3: UV/Vis spectra of **AP-4** in PBS and DMEM solutions.

Figure S4: UV/Vis spectra of **AP-5** in PBS and DMEM solutions.

Figure S5: Experimental FT-IR spectrum of **AP-5**.

Figure S6: Geometries of species involved in the hydrolysis of **AP-5** with one or two water molecules included in the calculation.

Figure S7: The calculated B3LYP ZPE energy profiles for the aquation process of **AP-1**, **AP-5**, and **AP-4** with one, two, and three water molecules included in the calculation

Table S1. Selected Bond Lengths (Å) for **AP-4** and **AP-5**.

**AP-4**		**AP-5**	
2.6143(3)	Pt1-I1	2.6239(4)	Pt1-I1
2.2843(4)	Pt1-As1	2.2823(6)	Pt1-As1
2.007(3)	Pt1-N1	2.007(5)	Pt1-N1
2.008(3)	Pt1-N2	2.005(5)	Pt1-N2
1.955(2)	As1-O1	1.971(4)	As1-O1
1.977(2)	As1-O2	1.959(4)	As1-O2
1.749(2)	As1-O3	1.742(3)	As1-O3
1.728(2)	As1-O4	1.742(3)	As1-O4
1.301(4)	N1-C4	1.286(7)	O1-C3
1.304(4)	O1-C4	1.300(8)	O2-C1
1.307(4)	O2-C1	1.324(9)	N1-C3
1.305(4)	N2-C1	1.296(7)	N2-C1
1.498(4)	C4-C5	1.497(8)	C1-C2
1.503(4)	C1-C2	1.493(8)	C3-C4
1.520(5)	C5-C6		
1.529(5)	C2-C3		

Table S2. Selected Bond Angles (°) for **AP-4** and **AP-5**.

**AP-4**		**AP-5**	
175.102(10)	As1-Pt1-I1	179.770(18)	As1-Pt1-I1
91.80(8)	N1-Pt1-I1	93.64(17)	N1-Pt1-I1
86.06(8)	N1-Pt1-As1	86.59(17)	N1-Pt1-As1
172.95(11)	N1-Pt1-N2	93.94(16)	N2-Pt1-I1
95.23(8)	N2-Pt1-I1	85.83(16)	N2-Pt1-As1
86.98(8)	N2-Pt1-As1	172.4(2)	N2-Pt1-N1
93.80(7)	O1-As1-Pt1	93.47(14)	O1-As1-Pt1
173.09(9)	O1-As1-O2	93.91(15)	O2-As1-Pt1
93.10(7)	O2-As1-Pt1	172.6(2)	O2-As1-O1
125.76(8)	O3-As1-Pt1	128.04(10)	O4-As1-Pt1
86.62(10)	O3-As1-O1	128.04 (10)	O3-As1-Pt1
89.24(10)	O3-As1-O2	87.30(13)	O3-As1-O1
128.88(8)	O4-As1-Pt1	87.30(13)	O4-As1-O1
86.34(10)	O4-As1-O1	122.0(6)	O1-C3-N1
122.5(3)	N2-C1-C2	116.7(6)	O1-C3-C4
110.7(3)	C1-C2-C3	121.4(6)	N1-C3-C4
114.9(3)	O1-C4-C5	88.16(13)	O4-As1-O2
89.42(10)	O4-As1-O2	88.15(13)	O3-As1-O2
105.30(11)	O4-As1-O3	103.9(2)	O3-As1-O4
116.50(19)	C4-O1-As1	116.9(4)	C3-O1-As1
116.61(19)	C1-O2-As1	116.2(4)	C1-O2-As1
121.7(2)	C4-N1-Pt1	121.1(4)	C3-N1-Pt1
121.2 (2)	C1-N2-Pt1	122.1(4)	C1-N2-Pt1
115.4(3)	O2-C1-C2	115.2(5)	O2-C1-C2
122.1(3)	N2-C1-O2	122.0(5)	N2-C1-O2
121.9 (3)	N1-C4-O1	122.8(6)	N2-C1-C2
123.2(3)	N1-C4-C5		
116.4(3)	C4-C5-C6		

Table S3. The most significant observed FT-IR and calculated frequencies with B3LYP method for **AP-4** [harmonic frequency (cm^{-1}), IR_{int} (K $mmol^{-1}$)].

Mode no.	Unscaled B3LYP	Scaled B3LYP	IR_{Int}[a]	IR exp. (cm^{-1})	Assignments [b]
81	3655	3538	81.3	3386	ν_{sym} O3–H + ν_{sym} O4–H
80	3653	3536	80.7	3356	ν_{asym} O3–H + ν_{asym} O4–H
79	3560	3446	45.1	3304	ν_{sym} N1–H + ν_{sym} N2–H
74	3101	3002	55.5	2966	ν_{asym} $C2H_2$ + ν_{asym} $C5H_2$ + ν_{asym} $C3H_3$ + ν_{asym} $C6H_3$
70	3032	2935	16.7	2934	ν_{sym} $C2H_2$ + ν_{sym} $C5H_2$
68	3031	2934	57.1	2877	ν_{sym} $C3H_3$ + ν_{sym} $C6H_3$
67	1571	1521	162.9	1567	νC1–N2 + νC4–N1
66	1553	1503	90.1	1552	ψ$C3H_3$ + ψ$C6H_3$
58	1465	1418	93.4	1445	νC1–O2 + νC4–O1 + βN1–H + βN2–H+ ω$C3H_3$+ ω$C6H_3$
56	1444	1398	43.0	1405	βN1–H + βN2–H + ω$C3H_3$+ ω$C6H_3$
51	1238	1198	60.5	1072	βN1–H + βN2–H + βC1–N2+ βC4–N1
45	1032	999	100.4	1042	βO3–H + βO4–H
43	1010	978	70.7	1008	βO3–H + βO4–H+ ω$C2H_2$+ ω$C5H_2$
42	1008	976	113.2	914	βN1–Pt–N2 + βC1–N2+ βC4–N1
41	910	881	36.8		βO1–C4–N1 + βO2–C1–N2
37	794	769	84.9	792	γN1–H + γN2–H
31	650	629	123.8	713	γO3–H + γO4–H + ν_{asym}As–O3 + ν_{asym}As–O4
30	645	625	155.6	693	νAs–Pt + ν_{sym}As–O3 + ν_{sym}As–O4
28	566	548	63.8	573	βC1–O2 + βC4–O2
24	384	372	9.6		νAs–Pt
18	248	240	22.9		βAs–Pt
14	201	195	13.8		γAs–Pt
13	200	194	1.9		νPt-I

[a]IR_{Int} – IR intensity; K $mmol^{-1}$; [b] ν – stretching; ν_{sym} symmetric stretching; ν_{asym} – asymmetric stretching; β – in-plane bending; γ – out-of-plane bending; ψ – scissoring; ω – wagging.

Table S4. Bond distances (Å) obtained at the theoretical level (in vacuum and water) and their experimental counterpart (R^2 correlation coefficient between DFT and XRD data).

**AP-4**				**AP-5**			
**Exp. (Å)**		**B3LYP (Å)**		**Exp. (Å)**		**B3LYP (Å)**	
		**Vacuum**	**Water**			**Vacuum**	**Water**
2.6143	Pt1-I1	2.6634	2.6952	2.6239	Pt1-I1	2.6633	2.6961
2.2843	Pt1-As1	2.378	2.3718	2.2823	Pt1-As1	2.3783	2.3711
2.007	Pt1-N1	2.0408	2.0469	2.007	Pt1-N1	2.0407	2.0467
2.008	Pt1-N2	2.0408	2.0469	2.005	Pt1-N2	2.0407	2.0467
1.955	As1-O1	2.0434	2.0254	1.971	As1-O1	2.0428	2.025
1.977	As1-O2	2.0434	2.0254	1.959	As1-O2	2.0428	2.025
1.749	As1-O3	1.8014	1.8024	1.742	As1-O3	1.8012	1.8024
1.728	As1-O4	1.8014	1.8024	1.742	As1-O3[i]	1.8012	1.8024
1.301	N1-C4	1.327	1.3323	1.286	O1-C3	1.3269	1.3329
1.304	O1-C4	1.327	1.3323	1.300	O2-C1	1.3269	1.3329
1.307	O2-C1	1.3154	1.3145	1.324	N1-C3	1.3149	1.314
1.305	N2-C1	1.3154	1.3145	1.296	N2-C1	1.3149	1.314
1.498	C4-C5	1.5069	1.5052	1.497	C1-C2	1.4993	1.4967
1.503	C1-C2	1.5069	1.5052	1.493	C3-C4	1.4993	1.4967
1.520	C5-C6	1.5394	1.5403				
1.529	C2-C3	1.5394	1.5403				
	R^2	0.9971	0.9983			0.9966	0.9978

Table S5. Comparison of bond angles (°) obtained by XRD and DFT (B3LYP) calculations in water and vacuum.

**AP-4**		B3LYP		**AP-5**		B3LYP	
Exp. (°)		Vacuum (°)	Water (°)	Exp. (°)		Vacuum (°)	Water (°)
175.10	As1-Pt1-I1	180.00	180.00	179.77	As1-Pt1-I1	180.00	180.00
91.80	N1-Pt1-I1	93.51	93.86	93.64	N1-Pt1-I1	93.50	93.84
86.06	N1-Pt1-As1	86.49	86.14	86.59	N1-Pt1-As1	86.51	86.15
173.0	N1-Pt1-N2	172.98	172.28	172.4	N2-Pt1-N1	173.01	172.31
95.23	N2-Pt1-I1	93.51	93.86	93.94	N2-Pt1-I1	93.49	93.85
86.98	N2-Pt1-As1	86.49	86.14	85.83	N2-Pt1-As1	86.51	86.15
93.80	O1-As1-Pt1	91.49	92.14	93.5	O1-As1-Pt1	91.49	92.16
173.09	O1-As1-O2	177.01	175.73	172.6	O2-As1-O1	177.02	175.68
93.10	O2-As1-Pt1	91.49	92.14	93.9	O2-As1-Pt1	91.49	92.16
125.76	O3-As1-Pt1	127.56	127.57	128.0	O3-As1-Pt1	127.54	127.57
86.6	O3-As1-O1	88.87	89.15	87.3	O3-As1-O1	88.81	89.08
89.2	O3-As1-O2	89.31	88.25	88.2	O3-As1-O2	89.37	88.29
128.88	O4-As1-Pt1	127.56	127.57	128.0	O4-As1-Pt1	127.54	127.55
86.3	O4-As1-O1	89.31	88.25	87.3	O4-As1-O1	89.37	88.29
122.5	N2-C1-C2	122.34	122.64	122.8	N2-C1-C2	122.58	122.86
114.9	O1-C4-C5	115.80	115.83	116.7	O1-C3-C4	115.44	115.53
121.9	N1-C4-O1	121.86	121.53	122.0	O1-C3-N1	121.98	121.60
89.4	O4-As1-O2	88.87	89.15	88.2	O4-As1-O2	88.81	89.08
105.3	O4-As1-O3	104.88	104.87	103.9	O3-As1-O4	104.92	104.88
116.5	C4-O1-As1	117.16	117.33	116.9	C3-O1-As1	117.10	117.26
116.6	C1-O2-As1	117.16	117.33	116.2	C1-O2-As1	117.10	117.26
121.7	C4-N1-Pt1	122.77	122.85	121.1	C3-N1-Pt1	122.68	122.81
121.2	C1-N2-Pt1	122.77	122.85	122.1	C1-N2-Pt1	122.68	122.81
115.4	O2-C1-C2	115.80	115.83	115.2	O2-C1-C2	115.44	115.53
122.1	N2-C1-O2	121.86	121.53	122.0	N2-C1-O2	121.98	121.60
123.2	N1-C4-C5	122.34	122.64	121.4	N1-C3-C4	122.59	122.86
110.7	C1-C2-C3	107.25	107.44				
116.4	C4-C5-C6	112.91	112.86				

Table S6. Natural bond orbital charge (e-) for **AP-1**, **AP-4,** and **AP-5**.

	**AP-1**			**AP-5**			**AP-4**	
	**Vacuum**	**Water**		**Vacuum**	**Water**		**Vacuum**	**Water**
Pt1	0.02607	0.08277	Pt1	-0.10053	-0.05198	Pt1	-0.10076	-0.05235
As1	2.08103	2.08417	As1	2.02631	2.04134	As1	2.02721	2.04207
Cl1	-0.41015	-0.50449	I1	-0.25657	-0.34452	I1	-0.25725	-0.34451
O1	-0.7813	-0.78702	O1	-0.77227	-0.78741	O1	-0.77658	-0.78969
O2	-0.78131	-0.78702	O2	-0.77227	-0.78741	O2	-0.77658	-0.78969
O3	-0.99748	-1.01308	O3	-0.98028	-1.00349	O3	-0.98047	-1.00375
O4	-0.99748	-1.01309	O4	-0.98028	-1.00348	O4	-0.98047	-1.00376
N1	-0.68445	-0.692	N1	-0.67995	-0.68445	N1	-0.68009	-0.68444
N2	-0.68445	-0.692	N2	-0.67995	-0.68444	N2	-0.68009	-0.68444
C1	0.66217	0.6721	C1	0.65897	0.66920	C1	0.66348	0.6732
C2	-0.63077	-0.63348	C2	-0.63174	-0.63529	C2	-0.43116	-0.43343
C3	0.66218	0.6721	C3	0.65897	0.66921	C4	0.66348	0.6732
C4	-0.63077	-0.63348	C4	-0.63174	-0.63529	C5	-0.43116	-0.43343
						C3	-0.56239	-0.56202
						C6	-0.56239	-0.56202
HO3	0.49625	0.50832	HO3	0.48825	0.50399	HO3	0.48789	0.50411
HO4	0.49624	0.50832	HO4	0.48825	0.50399	HO4	0.48789	0.50411
HN1	0.40405	0.40972	HN1	0.40171	0.41088	HN1	0.4015	0.41043
HN2	0.40405	0.40972	HN2	0.40171	0.41088	HN2	0.4015	0.41043
HC2	0.23767	0.24043	HC2	0.23747	0.24023	HC2	0.22918	0.23269
HC2	0.21191	0.22331	HC2	0.21037	0.22366	HC2	0.20307	0.21634
HC2	0.23347	0.24049	HC2	0.23286	0.24023			
HC4	0.23767	0.24043	HC4	0.23747	0.24023	HC4	0.22918	0.23269
HC4	0.21191	0.22331	HC4	0.21037	0.22366	HC4	0.20307	0.21634
HC4	0.23347	0.24049	HC4	0.23286	0.24023			
						HC3	0.20407	0.20908
						HC3	0.2094	0.20481
						HC3	0.19751	0.20007
						HC6	0.20407	0.20908
						HC6	0.2094	0.20481
						HC6	0.19751	0.20007

Stability and Solubility Studies of AP-4 and AP-5

a)

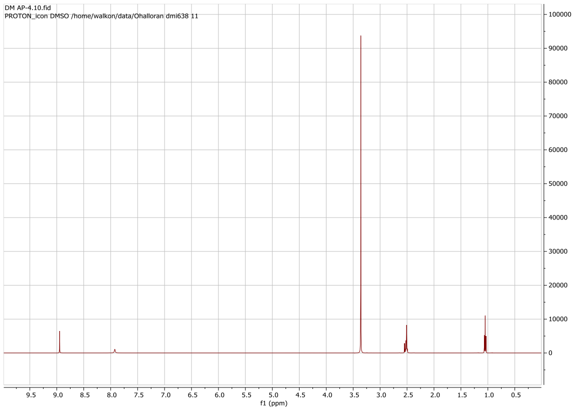

b)

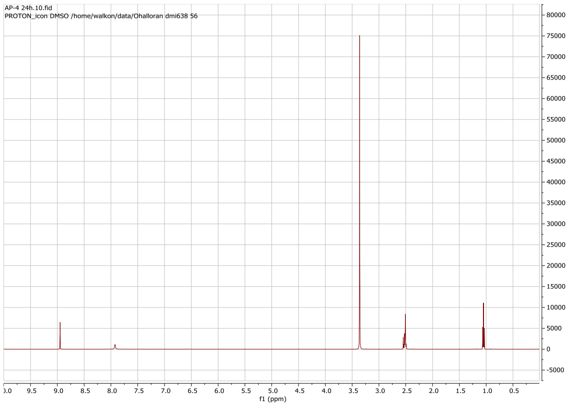

c)

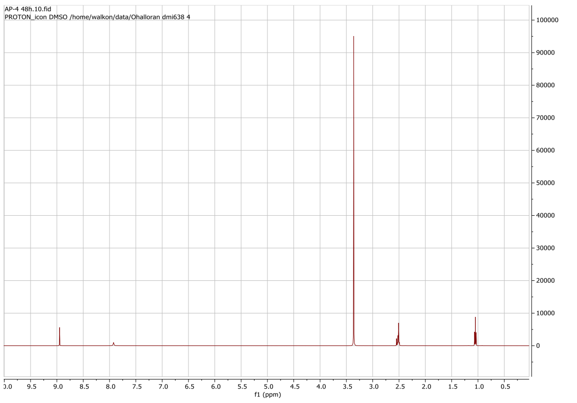

Figure S1. ^{1}H NMR spectra of **AP-4** acquired at 500 MHz in DMSO-d_6: a) immediately after dissolving; b) after 24 h; c) after 48 h. All spectra are recorded at room temperature (298.2 K). ^{1}H referenced to residual [D_5H]DMSO at 2.50 ppm. Chemical shifts in all spectra: 8.95 ppm (s, 2H-OH); 7.92 ppm (s, 2H-NH); ~ 2.50 ppm (4H-CH2) proton signal overlaps with the solvent signal; 1.05 ppm (s, 6H-CH_3).

a)

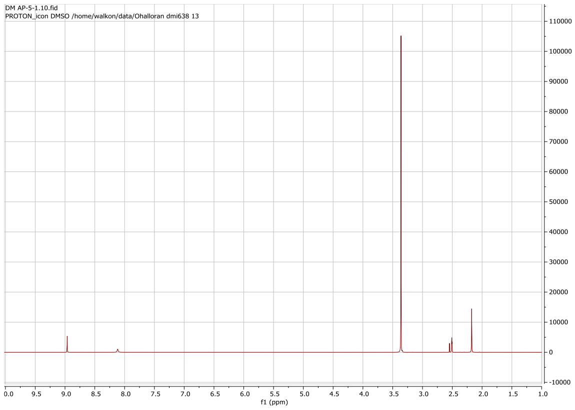

b)

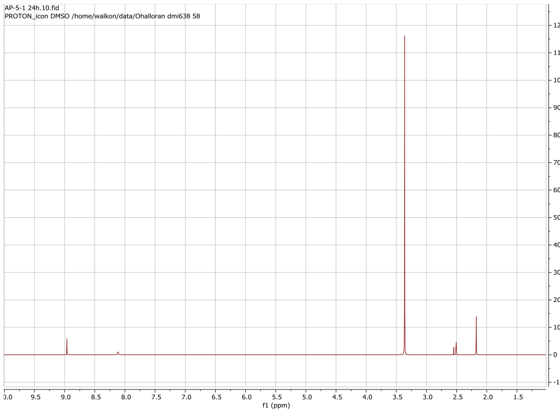

c)

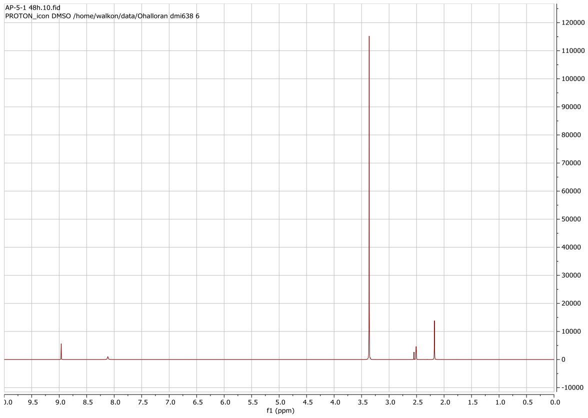

Figure S2. ^{1}H NMR spectra of **AP-5** acquired at 500 MHz in DMSO-d_6: a) immediately after dissolving; b) after 24 h; c) after 48 h. All spectra are recorded at room temperature (298.2 K). ^{1}H referenced to residual [D_5H]DMSO at 2.50 ppm. Chemical shifts in all spectra: 8.96 ppm (s, 2H-OH); 8.12 ppm (s, 2H-NH); 2.17 ppm (s, 6H-CH_3).

CH_3 signal (s) from $(CH_3)_2SO$ is present in all spectra in DMSO-d_6 at 2.55 ppm.

UV/Vis spectra of AP-4 in PBS and DMEM solutions

a)

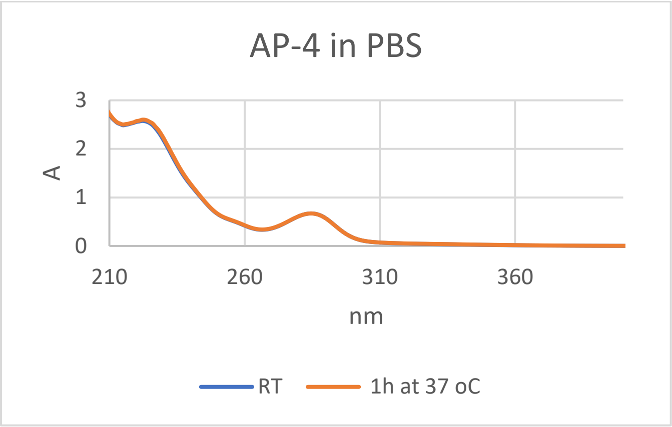

b)

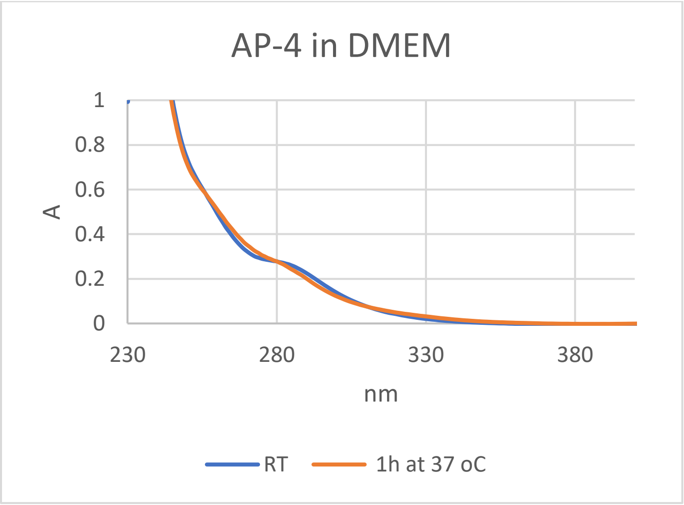

Figure S3. UV/Vis spectra evaluating the stability of **AP-4** in PBS (a) and DMEM (b) solutions.

AP-4 ($1.2x10^{-4}$ M) is stable in PBS (pH = 7.4) at room temperature and after 1h at 37 °C (**a**). In the DMEM solution ($1.2x10^{-4}$ M), the position of the first peak (~284 nm) is present as a shoulder. There is no significant difference in UV/Vis spectra of **AP-4** in DMEM solution (minus the phenol red indicator) at room temperature and after 1h at 37 °C. In contrast to PBS solutions, the DMEM media contains many additional biochemical supplements and these exhibit significantly more absorbance beginning at wavelengths below 280 nm. Thus, difference spectra are not useful below ca 240 nm.

All arsenoplatins have the position of the first peak at ~284 nm and a shoulder at ~250 nm. The substitution of a ligand bound to platinum (Cl^-, I^-, SCN^-) changes the position of the maximum at a shorter wavelength. For example, λ_1 in **AP-1** is at 283 nm and λ_1 in **AP-5** is at 283 nm; λ_2 in **AP-1** is at 240 nm and λ_2 in **AP-5** is at 225 nm (in PBS).

UV/Vis spectra of AP-5 in PBS and DMEM solutions

a)

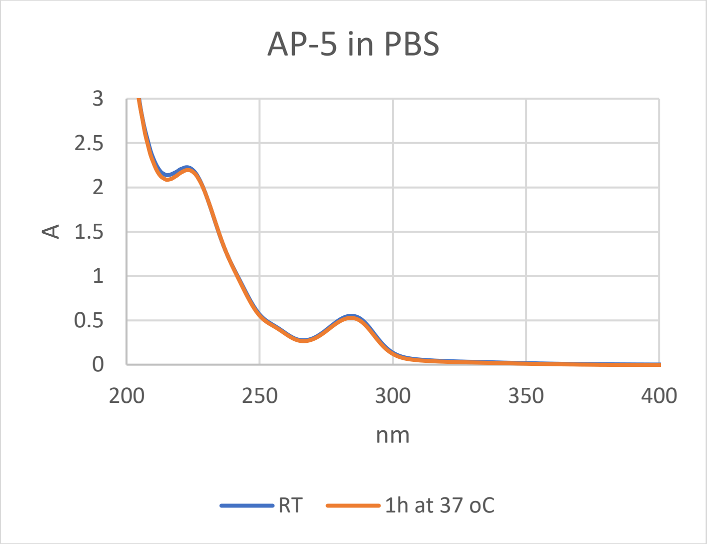

b)

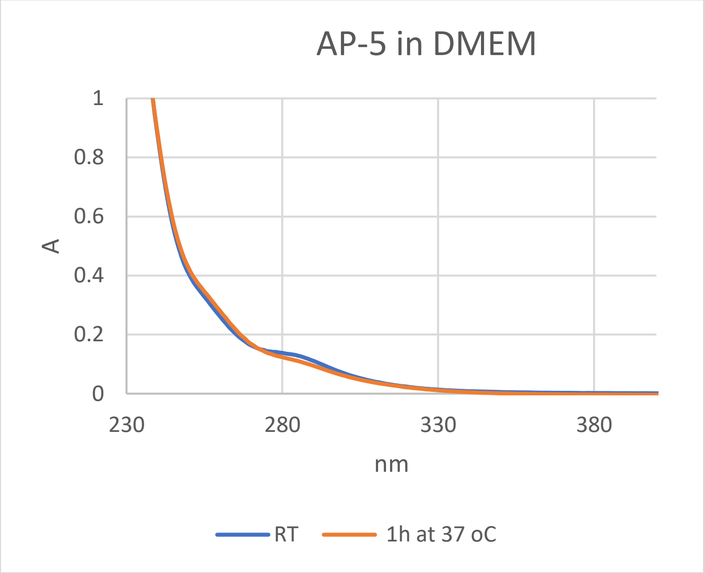

Figure S4. UV/Vis spectra evaluating the stability of AP-5 in (a) PBS and (b) DMEM solutions.

AP-5 ($1.4x10^{-4}$ M solution) is stable in PBS (pH = 7.4) at room temperature and after 1h at 37 °C (**a**). In DMEM ($1.4x10^{-4}$ M) solution (**b**), the first peak (~284 nm) is visible as a shoulder. There is no significant difference in UV/Vis spectra of **AP-5** in DMEM solution (minus the phenol red indicator) at room

temperature and after 1h at 37 °C. In contrast to PBS solutions, the DMEM media contains many additional biochemical supplements and these exhibit significantly more absorbance beginning at wavelengths below 280 nm. Thus difference spectra are not useful below ca 240 nm.

FT-IR Spectroscopy

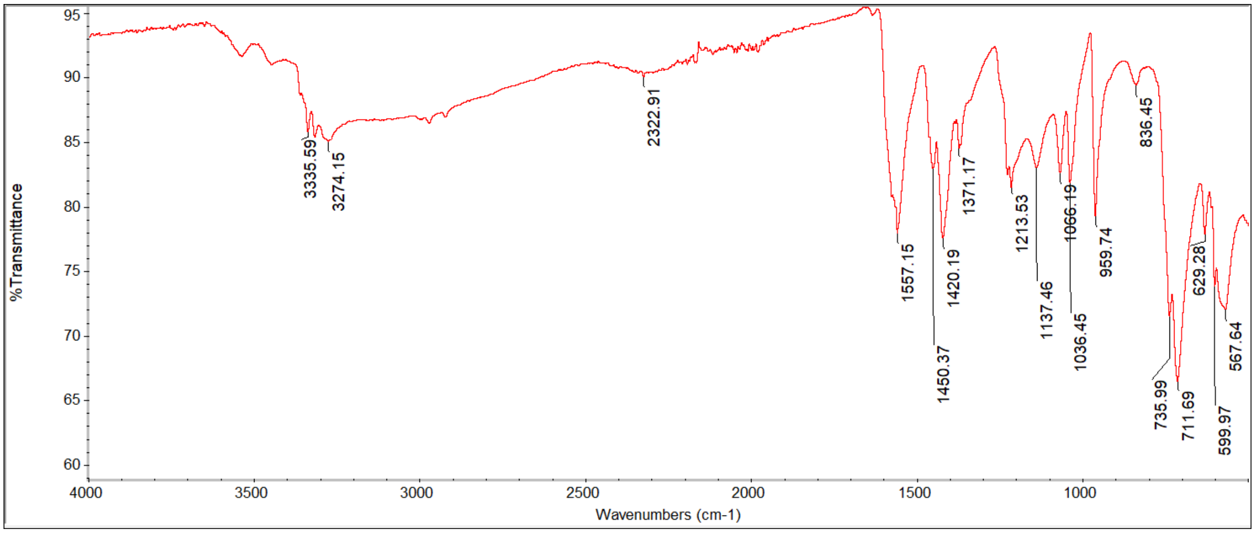

Figure S5. Experimental FT-IR spectrum of **AP-5** with two water molecules in the crystal lattice.

DFT calculations

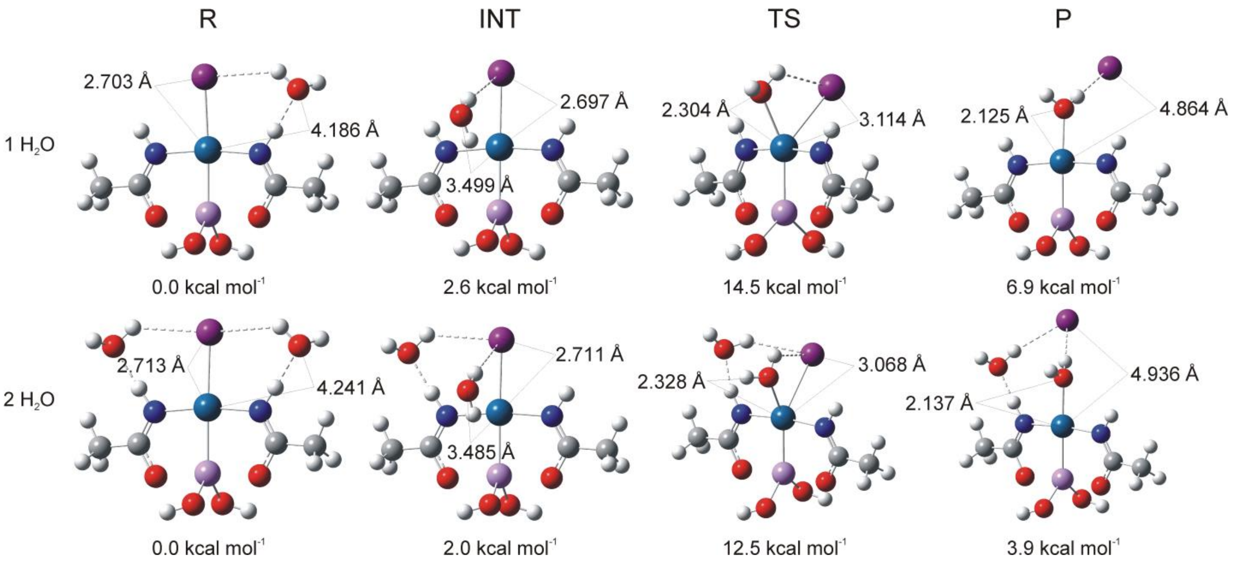

Figure S6. Geometries of species involved in the hydrolysis of **AP-5** with one or two water molecules included in the calculation. All energies are relative to the most stable reactant and are given in kcal/mol. (R = reactant, INT = intermediate, TS = transition state, P = product)

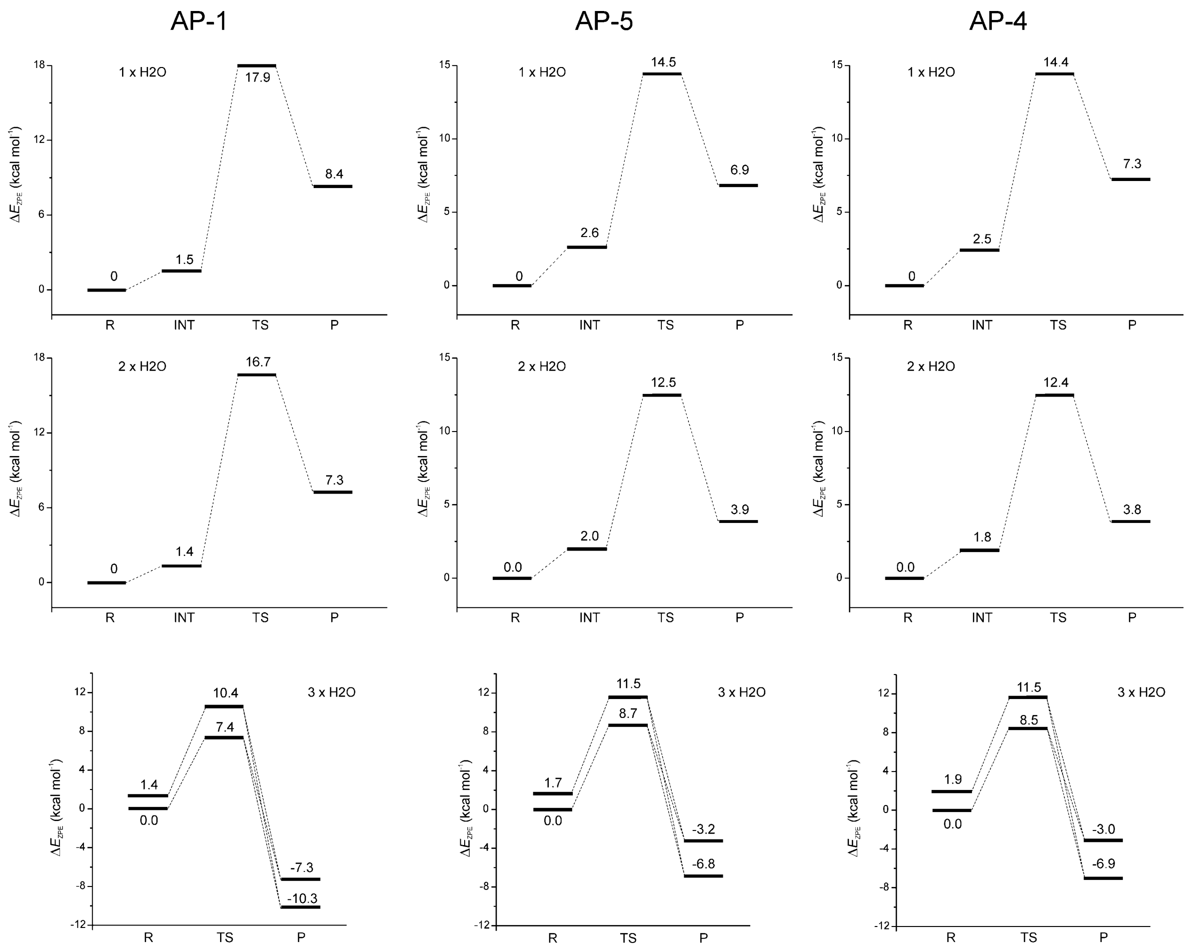

Figure S7. The calculated B3LYP ZPE energy profiles for the aquation process of **AP-1**, **AP-5**, and **AP-4** with one, two, and three water molecules included in the calculation. Energies are in kcal/mol and are relative to the most stable reactant. (R = reactant, INT = intermediate, TS = transition state, P = product).
